# Increasing Discards as an Unintended Consequence of Recent Changes in United States Kidney Allocation Policy

**DOI:** 10.1016/j.ekir.2023.02.1081

**Published:** 2023-02-25

**Authors:** Sumit Mohan, Miko Yu, Kristen L. King, S. Ali Husain

**Affiliations:** 1 Division of Nephrology, Department of Medicine, Vagelos College of Physicians and Surgeons, Columbia University, New York, New York, USA; 2 Columbia University Renal Epidemiology Group, New York, New York; 3 Department of Epidemiology, Mailman School of Public Health, Columbia University, New York, New York, USA

**Keywords:** deceased donor discards, kidney allocation, kidney transplant

Kidney transplantation is the preferred treatment for end-stage kidney disease.^1^ Despite a shortage of donated kidneys, the United States has the highest proportion of deceased donor kidneys (DDKs) recovered for transplant that are subsequently discarded; thereby resulting in the federal government prioritizing the improvement of organ utilization.^2^^,^^3^ Simultaneously, there is increased emphasis on improving equity in transplant access, including a revised allocation system (KAS250) in March 2021 that removed strict geographic boundaries previously used in local organ allocation in an effort to reduce geographic heterogeneity in transplant rates.^4^ However, KAS250 dramatically increased the complexity of interactions between transplant centers and organ procurement organizations,^5^ and the impacts of this change on organ utilization are not yet understood.

Among these 96,834 DDKs recovered during the study period, 21,411 were discarded (22%), including 13,229 of 64,281 (21%) pre-KAS250 and 8182 of 32,553 (25%) with KAS250 (*P* = 0.004), an increase that occurred despite similar overall quality of recovered DDKs in both eras (median kidney donor profile index 48% vs. 51%). DDK discard has increased across all adult donor age groups (18–60 years: 16% vs. 21%; ≥60 years: 54% vs. 60%, Supplementary Figure S1), among DDKs with medium (18% vs. 22%) and high kidney donor profile index (64% vs. 68%; Figure 1a), and for DDKs from donors after both cardiac death (22% vs. 31%) and brain death (20% vs. 23%; Figure 1b). Discard of low kidney donor profile index DDKs was unchanged (3% vs. 3%). An interrupted time series analysis also demonstrates an accelerated increase in DDK discards after the policy change (Supplementary Figure S2).

**Figure 1:**
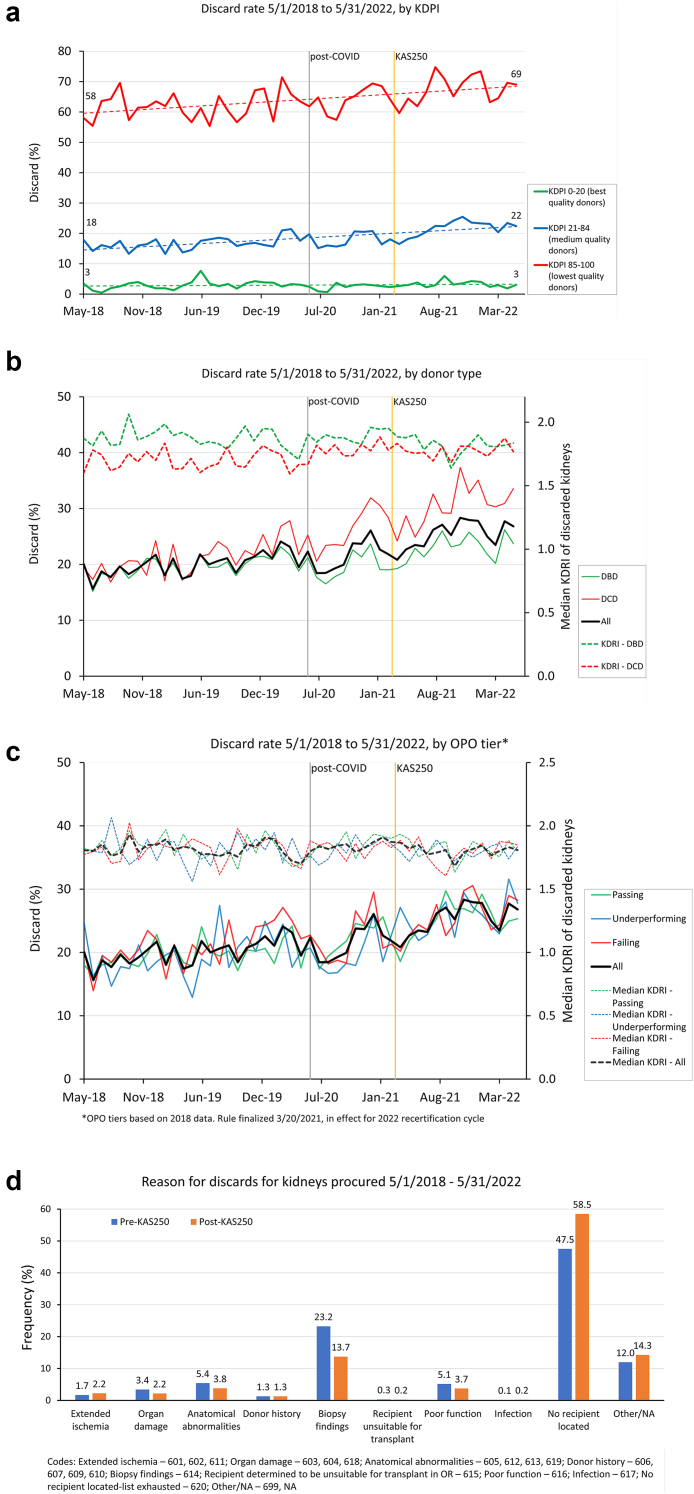
(a) Changes in United States deceased donor kidney utilization before and after the 2021 revision of the Kidney Allocation System by kidney donor profile index, (b) donation after cardiac death status, (c) and organ procurement organization performance status show increased organ discard, (d) with “No recipient located” predominating as the reason for discard.

DDK discard has also increased broadly among organ procurement organizations with high, medium, and low performance ratings (Figure 1c), indicating that this is not a geographic-specific or organ procurement organization-specific concern. The current discard rate of kidneys if present in prior years, after accounting for differences in discard rates for different quality organs, would result in 967 and 805 fewer transplants occurring in 2019 and 2020, respectively (Supplementary Table S1). Importantly, reasons for DDK discard also shifted with KAS250, with a greater proportion of discards attributable to the exhaustion of the list of eligible candidates without organ acceptance (Figure 1d), thereby supporting logistical complexity as a driver of worsened utilization.

Although the absolute number of DDK transplants has increased, over 1 in 4 DDKs recovered for transplant are not being transplanted, representing missed opportunities for hundreds of patients annually and increased health care system cost. This unintended deleterious consequence of the allocation system change requires urgent intervention to ensure that priceless organs are not wasted and that efforts to improve geographic equity in transplant do not come at a cost of worsening DDK utilization.

## Disclosure

All the authors declared no competing interests.

## Supplementary Material

**Supplementary Methods.**

**Figure S1.** Deceased donor kidney discard in the United States before versus after the 2021 update to the Kidney Allocation System by donor age group.

**Figure S2.** Interrupted time series comparing the rate of increase in deceased donor kidney discard in the United States before versus after the 2021 update to the Kidney Allocation System.

**Table S1.** Estimated increase in kidney discard in 2019 and 2020, based on the discard rate reflecting the 2021 update to the Kidney Allocation System.
