# Supplementary material for: Increasing Discards as an Unintended Consequence of Recent Changes in United States Kidney Allocation Policy
Source: Kidney Int Rep. 2023 Feb 25;8(5):1109–11. doi: 10.1016/j.ekir.2023.02.1081 (PMC10166727; doi:10.1016/j.ekir.2023.02.1081)
Supplement: Supplementary File (PDF) [file mmc1.pdf]

Supplementary Material

Supplementary Methods.

Supplemental Figure S1. Deceased donor kidney discard in the United States before versus after the 2021 update to the Kidney Allocation System by donor age group.

Supplemental Figure S2. Interrupted time series comparing the rate of increase in deceased donor kidney discard in the United States before versus after the 2021 update to the Kidney Allocation System. The regression is estimated using Newey-West standard errors with lag 0.

Supplemental Table S1. Estimated increase in kidney discard in 2019 and 2020, based on the discard rate reflecting the 2021 update to the Kidney Allocation System. The estimated increase in discard was calculated by assuming the median monthly discard rate for the observed number of recovered kidneys and subtracting the observed number of discarded kidneys within each year. For instance, assuming KDPI 21-84 organs had a discard rate of 22% in 2019 would have resulted in an excess of 763 kidney discards compared to the observed number of kidney discards within that group.

Supplementary Methods.

To understand the impact of KAS250 on DDK utilization, we performed a retrospective registry analysis using SRTR data (June 2022) with all kidneys recovered for transplantation from May 1, 2018 to May 31, 2022. Tier ratings (passing, underperforming, and failing) for 57 OPOs were published by CMS in 2020. Median Kidney Donor Risk Index (KDRI) and Kidney Donor Risk Index ([KDPI] 0-20, 21-84, and 85-100) were calculated based on the March 2022 OPTN mapping table. Monthly discard rates were calculated and stratified by donor age and type (death after cardiac death vs brain death). The 20 discrete reasons for discard identified in SRTR were mapped to ten overarching categories: (1) extended ischemia; (2) organ damage; (3) anatomical abnormalities; (4) donor history; (5) biopsy findings; (6) recipient determined to be unsuitable for transplant in OR; (7) poor function; (8) infection; (9) no recipient located – list exhausted; and (10) other/not available. Trends in monthly discard rates before and after introduction of KAS250 were analyzed using interrupted time series.

Supplemental Figure S1. Deceased donor kidney discard in the United States before versus after the 2021 update to the Kidney Allocation System by donor age group.

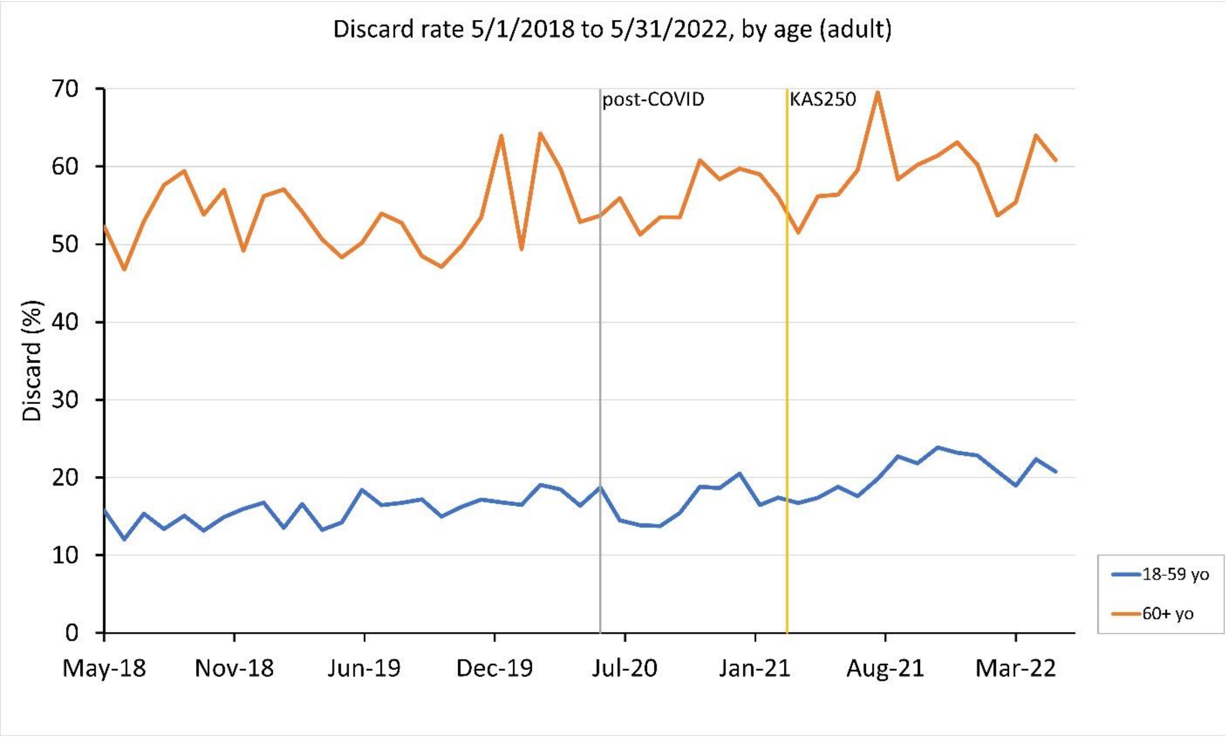

Supplemental Figure S2. Interrupted time series comparing the rate of increase in deceased donor kidney discard in the United States before versus after the 2021 update to the Kidney Allocation System. The regression is estimated using Newey-West standard errors with lag 0.

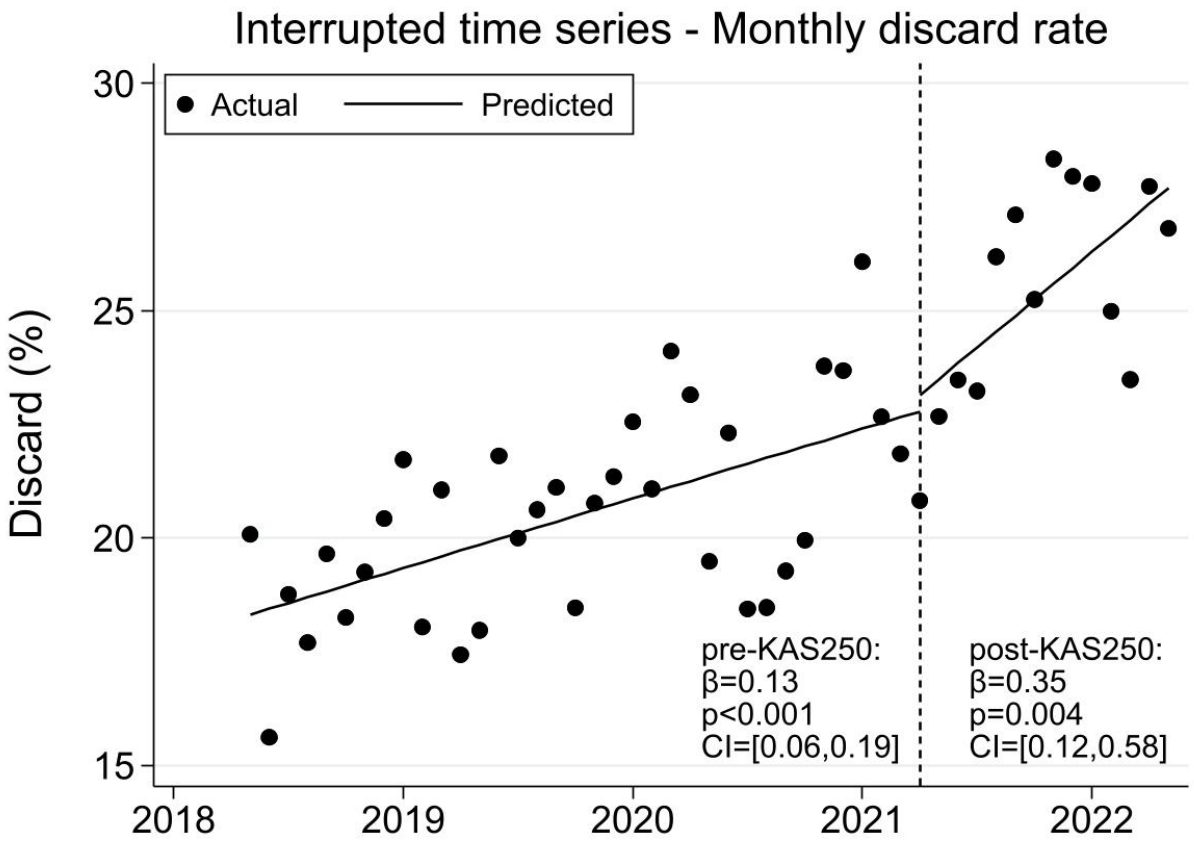

Supplemental Table S1. Estimated increase in kidney discard in 2019 and 2020, based on the discard rate reflecting the 2021 update to the Kidney Allocation System. The estimated increase in discard was calculated by assuming the median monthly discard rate for the observed number of recovered kidneys and subtracting the observed number of discarded kidneys within each year. For instance, assuming KDPI 21-84 organs had a discard rate of 22% in 2019 would have resulted in an excess of 763 kidney discards compared to the observed number of kidney discards within that group.

Post-KAS250 (April 2021 – May 2022)		2020		2019	
KDPI	Median monthly discard rate	Recovered (N)	Estimated increase in discard (N)	Recovered (N)	Estimated increase in discard (N)
0-20	3%	5,014	15	4,795	-12
21-84	22%	15,274	632	14,252	763
85-100	68%	3,450	158	3,159	216
Total	-	23,738	805	22,207	967
